# Obesity and thinness: insights from genetics

**DOI:** 10.1098/rstb.2022.0205

**Published:** 2023-10-23

**Authors:** Sadaf Farooqi

**Affiliations:** Wellcome-MRC Institute of Metabolic Science, Addenbrooke's Hospital, Box 289, Cambridge CB2 0QQ, UK

**Keywords:** genetics, obesity, thinness, leptin, melanocortin, hyperphagia

## Abstract

Genetic disruption of key molecular components of the hypothalamic leptin–melanocortin pathway causes severe obesity in mice and humans. Physiological studies in people who carry these mutations have shown that the adipose tissue-derived hormone leptin primarily acts to defend against starvation. A lack of leptin causes an intense drive to eat and increases the rewarding properties of food, demonstrating that human appetite has a strong biological basis. Genetic studies in clinical- and population-based cohorts of people with obesity or thinness continue to provide new insights into the physiological mechanisms involved in weight regulation and identify molecular targets for weight loss therapy.

This article is part of a discussion meeting issue ‘Causes of obesity: theories, conjectures and evidence (Part II)’.

## 1. Introduction

Susceptibility to weight gain within a permissive, obesogenic environment is influenced by genetic factors. Studies in monozygotic twins raised separately have shown that the heritability of body weight (the proportion of phenotypic variation explained by genetic variation) is at least 40–70% [1–3]. Additionally, longitudinal studies of Danish children who were adopted found they have body weights that are comparable to their biological rather than to their adoptive parents with whom they share the childhood environment [4,5]. Moreover, in studies of identical twins provided with excess calories, Bouchard and colleagues showed that members of a twin pair gained similar amounts of weight, indicating that genetic factors influence our response to the amount of food consumed [6]. Collectively, these studies have demonstrated that genetic factors influence body weight across the spectrum. We now know that genetic variation can cause severe obesity or increase the susceptibility to weight gain; similarly, there are variants that either protect against obesity or are associated with thinness [7].

**Figure 1.**
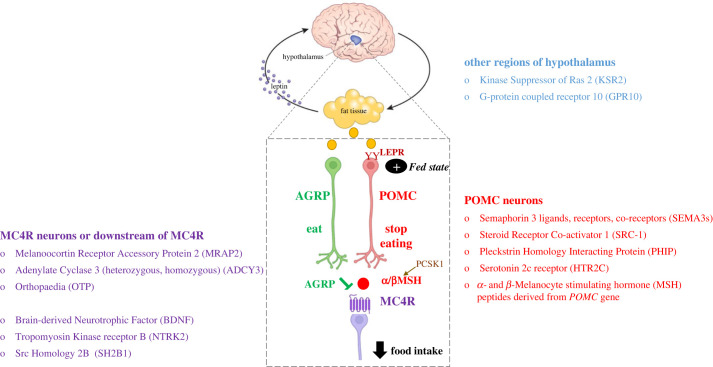
Genetic obesity syndromes affecting the leptin–melanocortin pathway. A schematic depicts the effects of leptin, a hormone released by adipose tissue, on neurons in the hypothalamus expressing the leptin receptor (LEPR). A fall in leptin activates neurons expressing Agouti Related Peptide (AGRP, green) to send the signal to eat in the fasted state. In the fed state, leptin stimulates neurons expressing Pro-opiomelanocortin (POMC, red). POMC is cleaved into smaller melanocortin peptides (α- and β-melanocyte stimulating hormone, MSH, red circles), which act as agonists at the Melanocortin 4 receptor (MC4R) expressed on downstream neurons (purple). Activation of MC4R sends the signal to decrease food intake. Pathogenic mutations and rare penetrant variants affecting the function of this circuit cause severe obesity.

To date, several different approaches have been used to identify the genes that regulate human body weight. Studies in children with severe obesity led to the identification of genetic obesity syndromes that display Mendelian inheritance [8]. Indeed, genetic testing for these conditions is now recommended as part of the clinical assessment of people with severe obesity that begins before the age of 5 years [9]. These genetic obesity syndromes predominantly affect the development and/or function of the leptin–melanocortin pathway, which plays a pivotal role in weight regulation (figure 1).

## 2. Leptin–melanocortin pathway and human energy homeostasis

Experimental studies in rodents showed that body weight is regulated by hypothalamic neurons that integrate hormonal signals from adipose tissue, such as leptin with short-term, meal-related neural and hormonal signals from the stomach and gastrointestinal tract including glucagon-like peptide-1 (GLP-1), Peptide YY and oxyntomodulin [10]. The physiological effects of leptin are mediated through the leptin receptor, which is highly expressed in the hypothalamus, midbrain, hippocampus and other brain regions [11]. In the arcuate nucleus of the hypothalamus, leptin stimulates the expression of pro-opiomelanocortin (POMC), a precursor peptide that is post-translationally processed to yield the melanocortin peptides α- and β-MSH (melanocyte stimulating hormone). In the pituitary gland, POMC is cleaved to yield adrenocorticotrophin (ACTH), which acts on the Melanocortin 2 receptor (MC2R) expressed on the adrenal gland to regulate production of cortisol. In the skin, melanocortin peptides regulate pigmentation by signalling through the Melanocortin 1 receptor (MC1R) and in the brain, α- and β-MSH activate signalling via MC4R to reduce food intake. In the fed state, leptin stimulates the expression of POMC and the firing of POMC neurons; POMC-derived peptides act as agonists at MC4R to decrease food intake [12]. At the same time, leptin suppresses the activity of adjacent neurons expressing Agouti Related Peptide, which antagonizes signalling at the MC3 and MC4 receptors. As these neurons project to and receive inputs from other brain regions, disruption of the leptin–melanocortin pathway can affect behaviour, neuroendocrine function and autonomic function.

## 3. Monogenic disorders cause severe obesity

Bi-allelic (homozygous or compound heterozygous) loss-of-function mutations in the genes encoding leptin and the leptin receptor cause hyperphagia, an intense drive to eat and severe obesity in the first year of life [13,14]. Administration of recombinant leptin to children with congenital leptin deficiency reversed hyperphagia and enhanced satiety, leading to substantial weight loss [15,16]. This work provided proof of principle that leptin is an essential regulator of human energy homeostasis and demonstrated that human eating behaviour is regulated by biological factors, rather than simply by volition. Leptin also regulates neural activation of dopaminergic neurons in mesolimbic brain regions to mediate the rewarding properties of food and drive food-seeking motivational behaviour in states of nutritional deprivation [17]. Leptin administration in congenital leptin deficiency reversed T cell-mediated immune dysfunction and permitted the onset of puberty at an appropriate developmental stage [16]. This work has shaped current understanding of how physiological states characterized by a fall in circulating leptin levels (starvation, the weight-reduced state), or by chronically low leptin levels (anorexia nervosa, disorders of adipose tissue development (lipodystrophies)), impact on immunity and reproduction.

Disruption of POMC and the enzyme that cleaves POMC, prohormone convertase 1 (PCSK1), also causes severe obesity with hypopigmentation (due to the loss of MC1R signalling) and cortisol deficiency (due to a lack of ACTH) [18,19]. Heterozygous loss-of-function mutations in MC4R are found in 5%–6% of patients with severe early onset obesity [20] and at a frequency of approximately 1/330 in the general UK population, making this the commonest gene in which variants contribute to obesity [21]. Heterozygous MC4R mutations are inherited in a co-dominant manner, with variable penetrance and expression [20]. The clinical features of MC4R deficiency closely mirror those seen in mice [22] and include hyperphagia, disproportionate hyperinsulinaemia, increased lean mass and increased linear growth [20,23]. Complete loss-of-function mutations have a larger impact on phenotype than partial loss-of-function mutations [24]. A subset of *MC4R* variants found at 1–2% minor allele frequency in the population increase the presence of MC4Rs at the plasma membrane by accelerated recycling to the membrane or reduced receptor internalisation. These gain-of-function *MC4R* variants are associated with substantial protection from obesity and type 2 diabetes, with a 50% reduction in risk in homozygous variant carriers [25]. These studies have highlighted the pivotal role of melanocortin tone in human weight regulation.

Alongside disorders that follow Mendelian inheritance, rare variants in multiple genes increase the risk of severe obesity in variant carriers. The characterization of these rare obesity-associated variants presents some challenges, but can be relevant for diagnostic and therapeutic purposes. For example, obesity-associated variants disrupt the secretion and/or function of 14 genes encoding Semaphorin-3 secreted proteins (*SEMA3A-G)*, their receptors (*NEUROPILIN-1/2)* and co-receptors (*PLXNA1–4*) involved in axon guidance [26]. Deletion of these genes in zebrafish increased somatic growth, body weight and/or percentage body fat and in mice, and *SEMA3*s acting via *NEUROPILIN-2* were shown to orchestrate the development of Pomc neuronal projections extending from the arcuate to the paraventricular nucleus of the hypothalamus. Another example is provided by the transcriptional co-activator Steroid Receptor Coactivator-1 (SRC-1), which modulates the ability of leptin to regulate the transcription of POMC in the hypothalamus by directly interacting with a target of leptin receptor activation, phosphorylated STAT3. This mechanism is disrupted by human *SRC-1* variants expressed in cells and in mice, where the acute anorectic response to leptin administration is impaired [27].

## 4. Conclusion

Cumulatively, genetic studies in people with severe obesity have delineated multiple molecular control points for melanocortin signalling, directly informing the development of new targets for weight loss therapy. The function of this pathway is also impaired in a number of pleiotropic obesity syndromes (for example, Bardet-Beidl syndrome and pseudohypoparathyroidism) where learning difficulties and organ-specific abnormalities predominate [28]. Informed by these mechanistic studies, successful phase 3 clinical trials have led to a second generation MC4R agonist being licensed in many countries for the treatment of several monogenic disorders (POMC, PCSK1, LEPR deficiencies and Bardet-Biedl Syndrome (BBS)) [29,30]. Clinical trials are ongoing in patients with rare penetrant variants in multiple other genes affecting the melanocortin pathway. Further work in cohorts with extreme phenotypes including persistent thinness is likely to provide insights into the mechanisms that regulate human body weight and may identify new targets for weight loss therapy.

## Data Availability

This article has no additional data.
